# Gleanings from the January Issues

**Published:** 1920-02-07

**Authors:** 


### THE HOSPITAL JOURNALS AND GAZETTES.

### Gleanings from the January Issues.

**St. Bartholomew's Hospital Journal.**—From the beginning of the current year an innovation takes place in the non-professional clinical units of the hospital. The posts of Medical and Surgical Registrars will no longer be in existence, but the place of these officers will be taken by chief assistants, one of whom will be attached to each unit. The duties of these chief assistants will consist in carrying out the registration of the firm, the performance of *post-mortem* examinations, supervision of the routine clinical and pathological work required in the investigations of the patients, and also to act as assistants in out-patient departments. These posts should prove extremely valuable, not only to the unit, but to the men themselves. It appears to be an advance in the right direction, for such men should go out well qualified in the particular branch they are working.

**St. Mary's Hospital Gazette.**—The Editor speaks of the tyranny of routine. Month after month, year after year, the same numbing round, the counterpart of every other, so that presently one day differs from another precisely as do prints taken from one negative—some a little darker than the rest. It is well from time to time to escape from this rut, to wear for a little an air of detachment, to laugh at oneself, and to view in its real proportions the measure of our small difficulties. It is so easy to get from a busy day the sense of doing things; to forget that when one has ceased to learn in out-patients, the power to teach has departed—if teaching be a vital thing, and not the remembrance of a few written tags complacently attached to appropriate patients—to lose sight of broad general principles in interminable committees; to become shop-sick and an apothecary. The happiest mortals have ever seemed to be they who, toiling diligently, can, when the day ends, join without bitterness in Puck's mockery, "Lord, what fools we mortals be."

**Guy's Hospital Gazette.**—The ancient remains discovered at Guy's during the process of excavation for a new wing are being examined in a businesslike way, and the fragments of pottery found are described in detail in the *Gazette*. These pieces belonged to dishes, bowls, etc., of the Roman household, and were mainly of the "terra sigillata" type, known formerly as Samian ware. This is characterised by being composed of fine-grained red clay—harder than the Arretine pottery of Italy—and by a fine red glaze like coral or sealing-wax. Most of the pottery was made at centres in Gaul, and the names of the potters stamped on the bases of pieces found in London are, in a large proportion of instances, identical with those found in excavations on the Continent. The two centres in Gaul where this pottery was known to have been manufactured were (1) near Rodez, in the Department of Aveyron, and (2) Lezoux, in the Puy de Dôme, Auvergne. The former pottery was made during the period A.D. 30, or even earlier, to A.D. 100, overlapping after A.D. 70 with that made at Lezoux, which remained in existence until A.D. 250. During the second century other potteries came into existence, notably at Rheinzabern, near Speyer, and lesser centres in Southern Bavaria. The remains unearthed at Guy's include specimens from all these localities.

**London (Royal Free Hospital) School of Medicine for Women.**—Patient (*brought in on ambulance with broken leg*): Well, doctor, it's broken, I think, because I heard the click; but now I comes to look, one of my trouser buttons is missing, and it may have been that."

Patient (*delighted at receiving an anonymous gift of two nightgowns*): "I think a white-robed angel must have sent me these."

Student: "Is the Belharzia case in this ward?" "No; there are no patients called Bell here." (After further inquiry): "And there are no heart cases in here either."

Patient: "I like that Scotch dressing, it draws out the 'umour."

Note from a Local Clergyman: "Have you two ladies (or students would do) who will teach bandaging to our girl guides?"

Excited Candidate (*immediately after examination*): "What would be the normal percentage of forks an ordinary G.P.I. would use?"

